# 3-bromopyruvate and buthionine sulfoximine effectively kill anoikis-resistant hepatocellular carcinoma cells

**DOI:** 10.1371/journal.pone.0174271

**Published:** 2017-03-31

**Authors:** Minjong Lee, Ara Jo, Seulki Lee, Jong Bin Kim, Young Chang, Joon Yeul Nam, Hyeki Cho, Young Youn Cho, Eun Ju Cho, Jeong-Hoon Lee, Su Jong Yu, Jung-Hwan Yoon, Yoon Jun Kim

**Affiliations:** 1 Department of Internal Medicine and Liver Research Institute, Seoul National University College of Medicine, Seoul, Korea; 2 Department of Internal Medicine, Kangwon National University Hospital, Chun-cheon, Korea; 3 University of Minnesota Hormel Institute, 801 16th Ave NE, Austin, MN, United States of America; Taipei Medical University, TAIWAN

## Abstract

**Background & aims:**

Acquisition of anoikis resistance is a prerequisite for metastasis in hepatocellular carcinoma (HCC). However, little is known about how energy metabolism and antioxidant systems are altered in anoikis-resistant (AR) HCC cells. We evaluated anti-tumor effects of a combination treatment of 3-bromopyruvate (3-BP) and buthionine sulfoximine (BSO) in AR HCC cells.

**Methods:**

We compared glycolysis, reactive oxygen species (ROS) production, and chemoresistance among Huh-BAT, HepG2 HCC cells, and the corresponding AR cells. Expression of hexokinase II, gamma-glutamylcysteine synthetase (rGCS), and epithelial–mesenchymal transition (EMT) markers in AR cells was assessed. Anti-tumor effects of a combination treatment of 3-BP and BSO were evaluated in AR cells and an HCC xenograft mouse model.

**Results:**

AR HCC cells showed significantly higher chemoresistance, glycolysis and lower ROS production than attached cells. Expression of hexokinase II, rGCS, and EMT markers was higher in AR HCC cells than attached cells. A combination treatment of 3-BP/BSO effectively suppressed proliferation of AR HCC cells through apoptosis by blocking glycolysis and enhancing ROS levels. In xenograft mouse models, tumor growth derived from AR HCC cells was significantly suppressed in the group treated with 3-BP/BSO compared to the group treated with 3-BP or sorafenib.

**Conclusions:**

These results demonstrated that a combination treatment of 3-BP/BSO had a synergistic anti-tumor effect in an AR HCC model. This strategy may be an effective adjuvant therapy for patients with sorafenib-resistant HCC.

## Introduction

For patients with advanced Hepatocellular carcinoma (HCC), only sorafenib significantly prolonged patient survival to date. However, the long-term survival benefit from sorafenib treatment is a modest improvement of 3 months, which is far from satisfactory [1]. Several other anti-angiogenesis drugs have been evaluated clinically for the treatment of HCC, but they were not satisfactory [2, 3]. Therefore, new strategies should be developed for patients with advanced HCC who did not respond to sorafenib or anti-angiogenesis agents.

Metastasis is a multistep process that includes dissociation of cancer cells from primary sites, survival in the vascular system, and proliferation in distant target organs. As a barrier to metastasis, cells normally undergo an apoptotic process known as “anoikis”, a form of cell death due to loss of contact with the extracellular matrix [4–6]. Cancer cells acquire anoikis resistance to survive after detachment from primary sites and travel through the circulatory and lymphatic systems to disseminate throughout the body [7].

The Warburg metabolic phenotype allows cancer cells to evade excessive reactive oxygen species (ROS) levels generated by mitochondrial respiration with NADPH generated by the pentose phosphate pathway (PPP), and therefore, cancer cells acquire a survival advantage by low oxidative stress when detached, contributing to anoikis resistance [8].

Anoikis-resistant (AR) cells enhance glycolysis for effective energy production under energy-limited conditions and activate antioxidant systems to prevent ROS-mediated apoptosis following detachment [8, 9]. Few studies have assessed the effects of a combination treatment of specific inhibitors of the Warburg effect and ROS suppression in AR HCC cells. In the glycolytic pathway, expression of hexokinase (HK) II, the rate-limiting enzyme in the first step, is significantly correlated with lactic acid production, which is the end product of glycolysis [10]. 3-BP is a structural analog of pyruvic acid. It is a strong alkylating agent toward HK II. The pyruvic group of 3-BP reacts with cysteine residue of HK II, reducing the activity of HK II [11]. HK II is both elevated in rapidly growing cancers and bound to mitochondrial voltage dependent anion channels (VDAC). When HK II is bound to VDAC, the HK II is not inhibited by glucose-6-phosphate. Therefore, ATP production is increased by accelerated glycolysis. In the cancer cell, 3-BP enters via monocarboxylate transporters (MCTs), which play a role for the efflux of lactic acid out of the normal cell. 3-BP uptake is particularly effective because of the overexpression of MCTs [12]. Previous studies reported that 3-BP exhibited high anticancer activity toward various cancers such as HCC, breast cancer, cervix cancer, colorectal cancer, endometrial cancer, gastric cancer, glioblastoma, kidney cancer, leukemia, lung cancer, lymphoma, melanoma, mesothelioma, multiple myeloma, neuroblastoma, ovarian cancer, pancreatic cancer, prostatic cancer, and squamous cell carcinoma [13].

Protection mechanism of cells against the detrimental effects of ROS is a reduced form of glutathione (GSH). Increased GSH level occurs in chemoresistance and/or radiation-resistant tumors to cope with increased ROS level: intracellular GSH levels are doubled in HCC compared to those in the normal liver [14–16]. Increased GSH levels in cancer cells are associated with higher levels of γ-glutamylcysteine synthetase (rGCS) and γ-glutamyl-transpeptidase (GGT) activities [15, 17]. It has been reported that GGT-overexpressing cells were more resistant to chemotherapeutic agents including 5-fluorouracil [18], cisplatin [19], and doxorubicin [20].

GSH depletion obtained by buthionine sulfoximine (BSO), the irreversible inhibitor of rGCS, is the most frequently used. Previous studies showed that BSO treatment is associated with many chemotherapeutic agents [21–25]. In different leukemia and lymphoma cells, it has been demonstrated that the death receptor-mediated apoptotic pathway induced by arsenic trioxide in combination with BSO, is triggered via c-Jun N-terminal kinase activation [26]. In melanoma cells, GSH depletion and GGT inhibition significantly increased cytotoxicity via oxidative stress [27]. In addition, in neuroblastoma cells susceptible to BSO treatment, DNA damage and cell apoptosis was occurred via ROS production and Protein kinase C-delta activation [28, 29]. Moreover, BSO plus melphalan was effective in treatment for patients with recurrent/refractory neuroblastoma [30]. Recently, it has been demonstrated that a combination of azathiopurine with BSO is useful for localized treatment of human HCC [31].

In this study, we aimed to clarify the characteristics of AR HCC cells derived from human HCC cell lines and examined HK II, rGCS expression, chemoresistance to sorafenib, invasion capabilities, and the anti-tumor effects of a combination treatment of 3-BP and BSO in AR HCC cells.

## Materials and methods

### Cell culture and reagents

The human HCC cell lines Huh-BAT and HepG2 cells were obtained from the Korea Cell Line Bank and cultured in Dulbecco’s modified Eagle’s medium (DMEM) (Life Technologies, Grand Island, NY) containing 10% fetal bovine serum. Cells were incubated at 37°C in a humidified atmosphere containing 5% CO_2_. For adherent cultures, HCC cells were grown in tissue culture dishes (Falcon, San Jose, CA), and for suspension cultures, cells were grown in dishes coated with 10 mg/ml Poly-hydroxyethylmethacrylate (Poly-HEMA) (Sigma, St. Louis, MO). To select cells that could survive in suspended culture, 1×10^6^ cells were seeded on Poly-HEMA-coated dishes and grown for 28 days. Fresh media were added every 3 days. After 7 days in culture, the cells were harvested and treated with diluted trypsin-EDTA (GibcoBRL, Grand Island, NY) to obtain a single-cell suspension for re-plating or MTS assay. We defined the suspended cells as anoikis-resistant cells; AR Huh-BAT and AR HepG2 cells were derived from the attached Huh-BAT and HepG2 cells, respectively (S1A Appendix in S1 Appendix).

### Western blot analysis

Human HCC cells were collected and lysed in lysis buffer (150 mM sodium chloride, 1% Triton X-100, 1% sodium deoxycholate, 0.1% sodium dodecyl sulfate, 50 mM Tris-HCl [pH 7.5], 2 mM EDTA, protease inhibitor cocktail). Then, the proteins were quantified with a BCA protein assay kit (Thermo, Rockford, IL). Equal amounts of protein were loaded onto a SDS-polyacrylamide gel (10% polyacrylamide) followed by electrophoresis at 100 V for 3 hours and were transferred to a polyvinylidene fluoride (PVDF) membrane at 50 V for 2 hours. The PVDF membrane was incubated at 4°C overnight with the target primary antibody. Each antibody used in this study was diluted in TBS-T (TBS/Tween 20: 2% skim milk) (S1B Appendix in S1 Appendix).

The secondary antibodies (horseradish peroxidase-conjugated anti-rabbit and mouse) were applied at room temperature for 1 hour. Immunoreactivity was developed using a peroxidase conjugate antiserum (Santa Cruz, Dallas, Texas) and detected by enhanced chemiluminescence reagents (Promega, WI). Western blotting of Huh-BAT, HepG2, AR Huh-BAT, and AR HepG2 cells treated with 40 μM of 3-BP (Sigma-Aldrich) alone, 200 μM of BSO (Santa Cruz, Dallas, Texas) alone, or 40 μM of 3-BP with 200 μM of BSO was performed.

### Invasion assay

For assessment of the invasion capability in AR HCC cells, invasion assays were performed using a cell invasion assay kit system with Boyden chambers (8 μm pore sizes, 24-well) (Merck Millipore, Billerica, MA). Confluent AR Huh-BAT and AR HepG2 cells were seeded in serum-free DMEM at a density of 10^5^ cells/upper chamber. AR Huh-BAT and AR HepG2 cells were treated as indicated (S1C Appendix in S1 Appendix).

### HCC xenograft mouse models

Five-week-old male BALB/c nude mice were purchased from Orient Bio (Gyeonggi-do, Korea). To generate tumors, 1×10^7^ Huh-BAT and AR Huh-BAT cells were subcutaneously inoculated into the right flank. After solid tumor formation, all mice bearing AR Huh-BAT cells were randomly divided into 4 groups, and each group consisted of 10 mice: (i) control (vehicle alone), (ii) sorafenib (once a day intraperitoneal injection of 10 mg/kg for 2 weeks), (iii) 3-BP (once a day intraperitoneal injection of 1.8 mg/kg for 2 weeks), and (iv) a combination treatment of 3-BP (two treatments per day and then one rest day, regimen repeatedly for 2 weeks with intraperitoneal injection of 1.8 mg/kg) and BSO (every other day, intraperitoneal injection of 250 mg/kg for 2 weeks).

The body weights of the mice were measured every other day with an electronic scale. Tumor size was measured every other day with an electronic caliper, and the volume was calculated by the following formula: tumor volume = (length × width × height) ÷ 2 [32, 33]. When the tumor volume reached a size of about 100 mm^3^, mice were treated with the agents: the maximum tumor size was 1,000 mm^3^ in the experiment. Mice were handled in maximum 5 per cage and ventilated cages with a 12-hour light/dark cycle. Animals received sterile food and water. Animals were monitored twice daily with health monitor forms and managed aseptically. None of mice showed sign of illness or died before the experimental endpoint in this study. We euthanized any animals deemed to be at humane endpoints: humane endpoints included one or more of the following: body weight loss of > 20% body weight, labored breathing, larger tumors > 1,000 mm^3^, tumor weight > 10% body weight, tumor ulcer necrosis, lack of response to stimulus and lethargic animal. For euthanasia, the inhalation method was performed by using 70% carbon dioxide. After finishing the treatment schedule, mice were anesthetized with isoflurane, and tissues and tumors were harvested for analysis.

To quantify tumor apoptosis, terminal deoxynucleotidyl transferase dUTP nick end labeling (TUNEL) staining was performed. All protocols for the animal experiments were reviewed and approved by the Institutional Animal Care and Use Committee of the Seoul National University Hospital (IACUC No. 14-0207-S1A1). All animal procedures were in accordance with the “Guide for the Care and Use of Laboratory Animals” issued by the Institute of Laboratory Animal Resources Commission on Life Science, US National Research Council.

Detailed information about additional materials and methods is provided: measurement of intracellular lactic acid levels (S1D Appendix in S1 Appendix), measurement of intracellular glutathione levels (S1E Appendix in S1 Appendix), Detection of intracellular ROS production (S1F Appendix in S1 Appendix), apoptosis determination (S1G Appendix in S1 Appendix), and statistical analysis (S1H Appendix in S1 Appendix).

## Results

### Induction of glycolysis and antioxidant systems by matrix detachment in AR HCC cells

We first evaluated change of enzyme expression related to glycolysis and antioxidant systems, lactic acid, GSH, ROS production after matrix detachment. After matrix detachment, glycolysis was potentiated with increase of antioxidant systems and decrease of ROS production.

**Fig 1:**
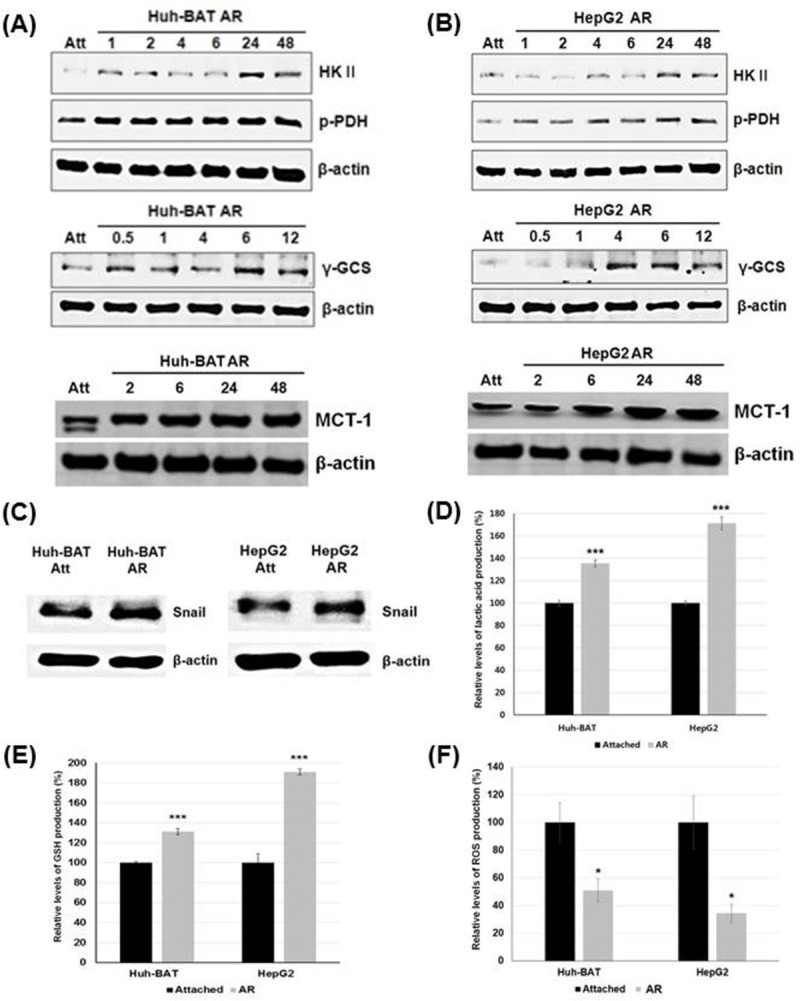
Changes in glycolysis, EMT markers, and ROS production after matrix detachment. (A) AR Huh-BAT cells had higher expression of HK II, p-PDH, rGCS, and MCT-1 compared to Huh-BAT and HepG2 cells at the indicated time points after matrix detachment. (B) AR HepG2 cells had higher expression of HK II, p-PDH, rGCS, and MCT-1 compared to Huh-BAT and HepG2 cells at the indicated time points after matrix detachment. (C) Expression of Snail was potentiated in AR Huh-BAT and AR HepG2 cells compared to Huh-BAT and HepG2 cells, respectively. (D) Lactic acid production in AR Huh-BAT and AR HepG2 cells was significantly increased compared to that in Huh-BAT and HepG2 cells, respectively. (E) Glutathione production in AR Huh-BAT and AR HepG2 cells was significantly increased compared to that in Huh-BAT and HepG2 cells, respectively. (F) Production of reactive oxygen species in AR Huh-BAT and AR HepG2 cells was significantly decreased compared to that in Huh-BAT and HepG2 cells, respectively. *P<0.05; ***P<0.001. Abbreviation: Att, attached; AR, anoikis-resistant; GSH, glutathione; HK II, hexokinase II; h, hours; MCT-1, monocarboxylate transporter-1; p-PDH, phosphorylated pyruvate dehydrogenase; rGCS, gamma-glutamylcysteine synthetase; ROS, reactive oxygen species.

AR Huh-BAT and AR HepG2 cells upregulated HK II, p-PDH, MCT-1, and rGCS compared to Huh-BAT and HepG2 cells (Fig 1A and 1B). Expression of Snail was increased 2 hours after matrix detachment in AR Huh-BAT and AR HepG2 cells compared to Huh-BAT and HepG2 cells (Fig 1C).

Intracellular lactic acid and GSH levels in AR Huh-BAT and AR HepG2 cells were significantly increased compared to those in Huh-BAT and HepG2 cells (*P*<0.001, AR Huh-BAT cells; *P*<0.001, AR HepG2 cells) (Fig 1D and 1E). Intracellular ROS levels in AR Huh-BAT and AR HepG2 cells were significantly decreased compared to Huh-BAT and HepG2 cells (*P*<0.01, AR Huh-BAT cells; *P*<0.05, AR HepG2 cells) (Fig 1F).

### Chemoresistance and tumor growth rates in AR HCC cells

To evaluate chemoresistance in AR HCC cells, cell viabilities were measured when AR HCC cells were exposed to various chemo-agents. Cell viabilities of AR HCC cells were compared to those of attached HCC cells. AR HCC cells showed chemoresistance to conventional chemo-agents, particularly sorafenib.

**Fig 2:**
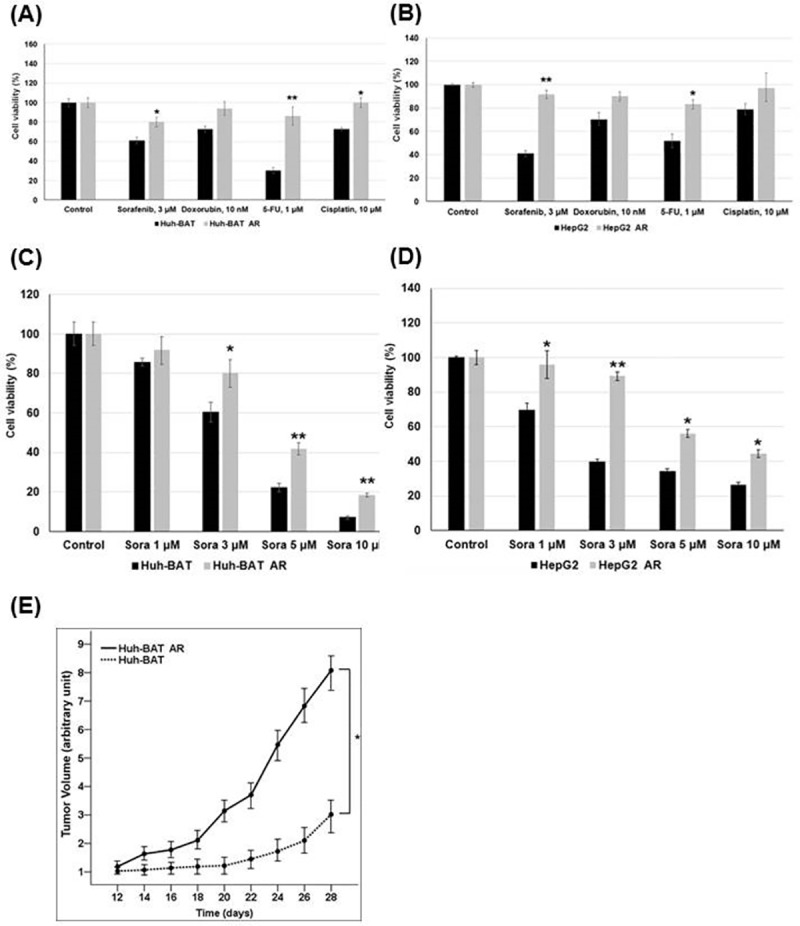
Chemoresistance phenotype of the AR HCC cells compared to attached HCC cells. (A) AR Huh-BAT cells had significantly higher viabilities at 3 μM of sorafenib, 1 μM of 5-FU, and 10 μM of cisplatin compared to Huh-BAT cells. (B) AR HepG2 cells had significantly higher viabilities at 3 μM of sorafenib and 1 μM of 5-FU compared to HepG2 cells. (C) AR Huh-BAT cells had significantly higher viabilities at concentrations of 3, 5, and 10 μM of sorafenib compared to Huh-BAT cells. (D) AR HepG2 cells had significantly higher viabilities at concentrations of 1, 3, 5, and 10 μM of sorafenib compared to HepG2 cells. (E) Tumor growth rates of AR Huh-BAT cells were significantly higher than those of Huh-BAT cells in xenograft nude mice. *P<0.05; **P<0.01. Abbreviation: AR, anoikis-resistant; sora, sorafenib; 5-FU, 5-fluorouracil.

AR Huh-BAT and AR HepG2 cells showed significantly higher viabilities at each concentration of sorafenib, doxorubicin, 5-FU, and cisplatin for 48 hours exposure than Huh-BAT and HepG2 cells; AR Huh-BAT and AR HepG2 cells showed chemoresistance (*P*<0.05, sorafenib; *P*<0.01, 5-FU; *P*<0.05, cisplatin in AR Huh-BAT cells and *P*<0.01, sorafenib; *P*<0.05, 5-FU in AR HepG2 cells) (Fig 2A and 2B).

After sorafenib treatments, the viabilities of the AR Huh-BAT and AR HepG2 cells were significantly different from those of the Huh-BAT and HepG2 cells. At high doses of sorafenib (5 and 10 μM), AR Huh-BAT and AR HepG2 cells showed significantly higher viabilities than Huh-BAT and HepG2 cells (*P*<0.01 at 5 μM sorafenib; *P*<0.01 at 10 μM sorafenib in AR Huh-BAT cells and *P*<0.05 at 5 μM sorafenib; *P*<0.05 at 10 μM sorafenib in AR HepG2 cells) (Fig 2C and 2D).

The tumor growth rates of AR Huh-BAT cells were increased compared to those of Huh-BAT cells. Tumors derived from AR Huh-BAT cells in a mouse xenograft model grew significantly faster than those from Huh-BAT cells (*P*<0.05) (Fig 2E).

### Changes of intracellular ROS and lactic acid levels by 3-BP, BSO, and a combination treatment of 3-BP and BSO

We evaluated change of glycolysis and ROS production after treatment of 3-BP and BSO: to clarify whether BSO can potentiate glycolysis or not, and 3-BP can potentiate ROS production or not. BSO significantly potentiated glycolysis and 3-BP also increased oxidative stress in AR HCC cells. It reflects that glycolysis was closely associated with ROS production in AR HCC cells and that BSO pre-treatment sensitized HCC cells to 3-BP treatment via increasing glycolysis.

**Fig 3:**
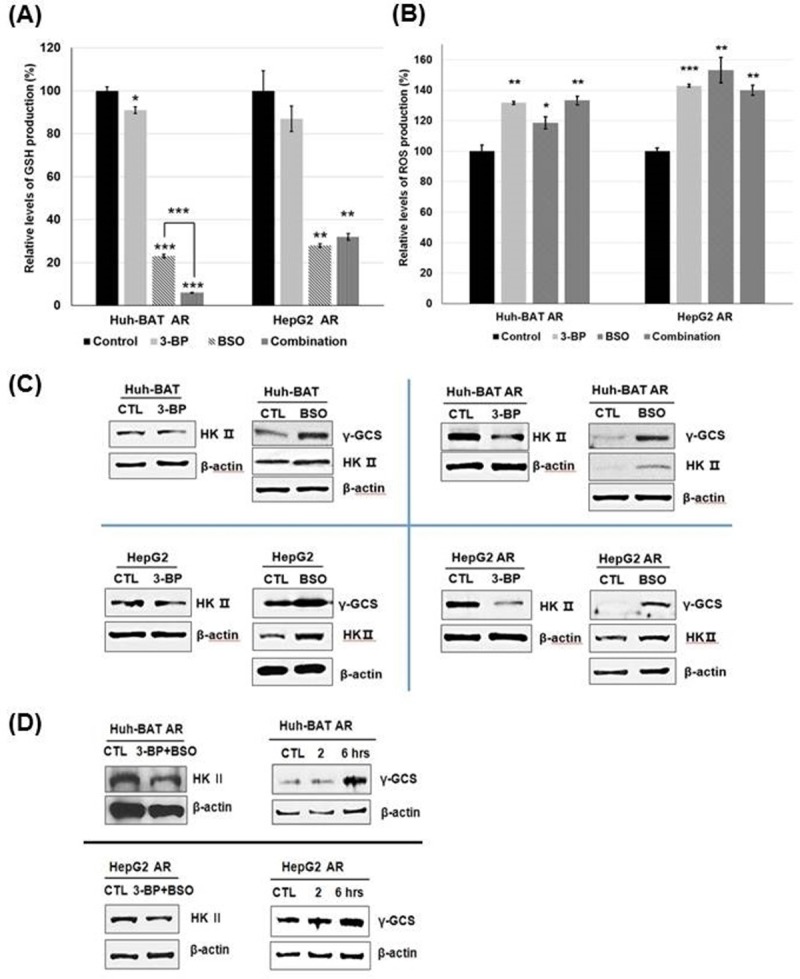
Changes of ROS and glutathione levels following 3-BP, BSO, or a combination treatment. (A) GSH production was significantly suppressed after BSO or a combination treatment of 3-BP and BSO compared to the control in AR Huh-BAT and AR HepG2 cells. (B) ROS production was significantly increased by 3-BP, BSO, and a combination treatment of 3-BP and BSO compared to the control. (C) Expression of HK II was decreased after 3-BP treatment and increased after BSO treatment. Expression of rGCS was increased after BSO treatment. (D) Following treatment with a combination of 3-BP and BSO, HK II expression was suppressed, and rGCS expression was increased compared to the control. Each assay and western blotting were performed at condition of 3-BP treatment 40 μM, 12 hour; BSO 200 μM, 24 hours; a combination treatment at 3-BP 40 μM, 12 hours after 24 hours pre-exposure of BSO 200 μM. *P<0.05; **P<0.01; ***P<0.001. Abbreviation: AR, anoikis-resistant; BSO, buthionine sulfoximine; CTL, control; GSH, glutathione; HK II, hexokinase II; rGCS, gamma-glutamylcysteine synthetase; ROS, reactive oxygen species; 3-BP, 3-bromopyruvate.

After BSO treatments, intracellular ROS levels were increased via suppression of GSH levels. 3-BP also significantly increased intracellular ROS levels. However, 3-BP did not effectively suppress intracellular GSH levels. BSO treatment and a combination treatment of 3-BP with BSO significantly suppressed intracellular GSH production (*P*<0.001, BSO; *P*<0.001, a combination treatment in AR Huh-BAT cells and *P*<0.01, BSO; *P*<0.01, a combination treatment in AR HepG2 cells) (Fig 3A). Intracellular ROS production in AR Huh-BAT and AR HepG2 cells was significantly increased after BSO, 3-BP, and a combination treatment of 3-BP and BSO compared to the control (*P*<0.01, 3-BP; *P*<0.05, BSO; *P*<0.01, a combination treatment in AR Huh-BAT cells and *P*<0.001, 3-BP; *P*<0.05, BSO; *P*<0.01, a combination treatment in AR HepG2 cells) (Fig 3B). At incubation time of 48 hours of 3-BP after 24 hours of BSO pre-treatment, intracellular glutathione levels were close to zero levels in AR Huh-BAT and AR HepG2 cells. In AR Huh-BAT and AR HepG2 cells treated with BSO for 24 hours, expression of rGCS was potentiated (Fig 3C). A combination treatment of 3-BP and BSO in AR Huh-BAT and AR HepG2 cells induced rGCS expression via negative feedback in response to the increased ROS levels (Fig 3D).

### Changes of intracellular lactic acid levels by 3-BP, BSO, and a combination treatment of 3-BP and BSO

**Fig 4:**
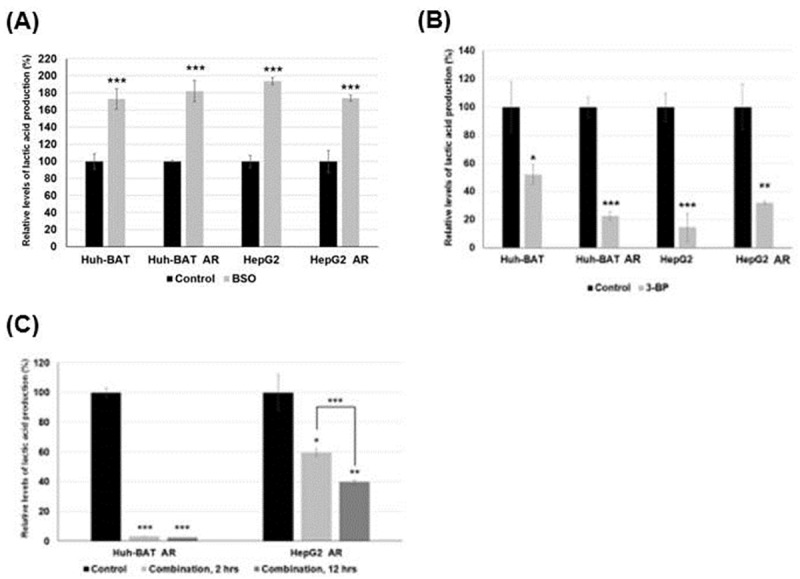
Changes of lactic acid levels following 3-BP, BSO, or a combination treatment. (A) Lactic acid production in Huh-BAT, HepG2, and the corresponding AR cells was significantly increased after BSO treatment compared to the control. (B) Lactic acid production in Huh-BAT, HepG2, and the corresponding AR cells was significantly suppressed after 3-BP treatment compared to the control. (C) Lactic acid production was significantly suppressed after a combination treatment of 3-BP and BSO in AR Huh-BAT and AR HepG2 cells; there was a significant difference among baseline, 2, and 12 hours exposure to the combination treatment. Each assay and western blotting was performed at condition of 3-BP treatment 40 μM, 12 hour; BSO 200 μM, 24 hours; a combination treatment at 3-BP 40 μM, 12 hours after 24 hours pre-exposure of BSO 200 μM. *P<0.05; **P<0.01; ***P<0.001. Abbreviation: AR, anoikis-resistant; BSO, buthionine sulfoximine; CTL, control; HK II, hexokinase II; rGCS, gamma-glutamylcysteine synthetase; ROS, reactive oxygen species; 3-BP, 3-bromopyruvate.

BSO treatment stimulated lactic acid production, and 3-BP suppressed lactic acid production. A combination treatment of 3-BP and BSO effectively suppressed lactic acid production. Lactic acid production in Huh-BAT, HepG2 cells, and the corresponding AR cells was significantly increased 48 hours after BSO treatment (*P*<0.001 for Huh-BAT cells; *P*<0.001 for AR Huh-BAT cells; *P*<0.001 for HepG2 cells; *P*<0.001 for AR HepG2 cells) (Fig 4A). 3-BP treatment significantly suppressed lactic acid production in these cells compared to the control (*P*<0.05 in Huh-BAT cells; *P*<0.001 in AR Huh-BAT cells; *P*<0.001 in HepG2 cells; *P*<0.01 in AR HepG2 cells) (Fig 4B). Expression of HK II was increased when Huh-BAT, HepG2, and the corresponding AR cells were treated with BSO (Fig 3C). Expression of HK II was suppressed 2 hours after 3-BP treatment in Huh-BAT, HepG2, and the corresponding AR cells (Fig 3C).

A combination treatment of 3-BP and BSO significantly suppressed lactic acid production in AR Huh-BAT and AR HepG2 cells compared to the control (*P*<0.001 in AR Huh-BAT cells; *P*<0.05 in AR HepG2 cells) (Fig 4C). At incubation time of 48 hours of 3-BP after 24 hours of BSO pre-treatment, intracellular lactic acid levels were close to zero levels in all cell lines. A combination treatment of 3-BP and BSO suppressed HK II expression in AR Huh-BAT and AR HepG2 cells (Fig 3D).

### Synergistic anti-tumor effects of a combination treatment of 3-BP and BSO in AR HCC cells

**Fig 5:**
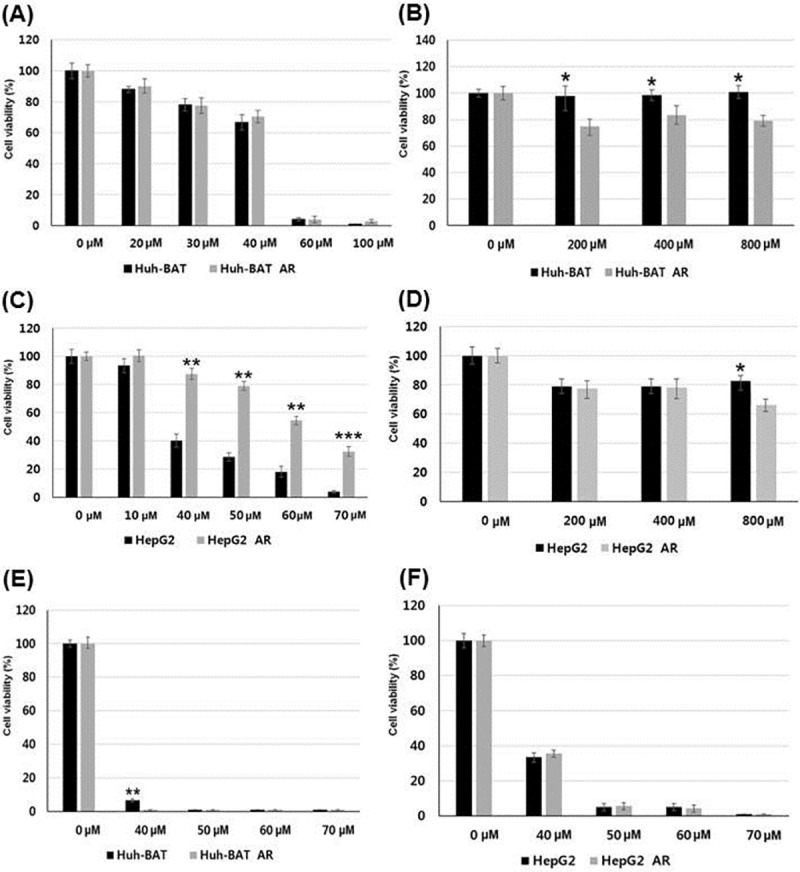
Cell viabilities of Huh-BAT, HepG2, and the corresponding AR cells after 3-BP, BSO, and a combination treatment. (A) Viabilities of Huh-BAT and AR Huh-BAT cells were decreased after 3-BP treatment. (B) In AR Huh-BAT cells, cell viabilities at each concentration were significantly lower after BSO treatment compared to those of Huh-BAT cells. (C) Viabilities of AR HepG2 cells were significantly higher than those of HepG2 cells after 3-BP treatment. (D) In HepG2 and AR HepG2 cells, cell viabilities were not effectively suppressed after BSO treatment. (E) Viabilities of Huh-BAT and AR Huh-BAT cells were effectively suppressed after a combination treatment of 3-BP and BSO. (F) Viabilities of HepG2 and AR HepG2 cells were effectively suppressed after a combination treatment of 3-BP and BSO. Abbreviation: AR, anoikis-resistant; BSO, buthionine sulfoximine; 3-BP, 3-bromopyruvate.

In Huh-BAT and AR Huh-BAT cells, 3-BP effectively inhibited cell proliferation at a concentration higher than 40 μM after 48 hours of exposure (Fig 5A). 3-BP suppressed cell proliferation in Huh-BAT and AR Huh-BAT cells. However, BSO did not effectively suppress cell proliferation even at high dose of 800 μM after 48 hours of exposure (Fig 5B). In HepG2 and AR HepG2 cells, 3-BP effectively inhibited cell proliferation at a concentration higher than 60 μM after 48 hours of exposure. AR HepG2 cells showed chemoresistance: significant higher viabilities than those of HepG2 cells (all, *P*<0.01) (Fig 5C). BSO did not effectively suppress cell proliferation at any doses (Fig 5D).

When attached and AR HCC cells were treated with a combination of 3-BP and BSO, cell viabilities were effectively suppressed at 3-BP concentrations higher than 40 μM with 24-hour pre-treatment of BSO (200 μM). A combination treatment effectively suppressed cell viabilities of Huh-BAT and AR Huh-BAT cells, HepG2 and AR HepG2 cells (Fig 5E and 5F).

**Fig 6:**
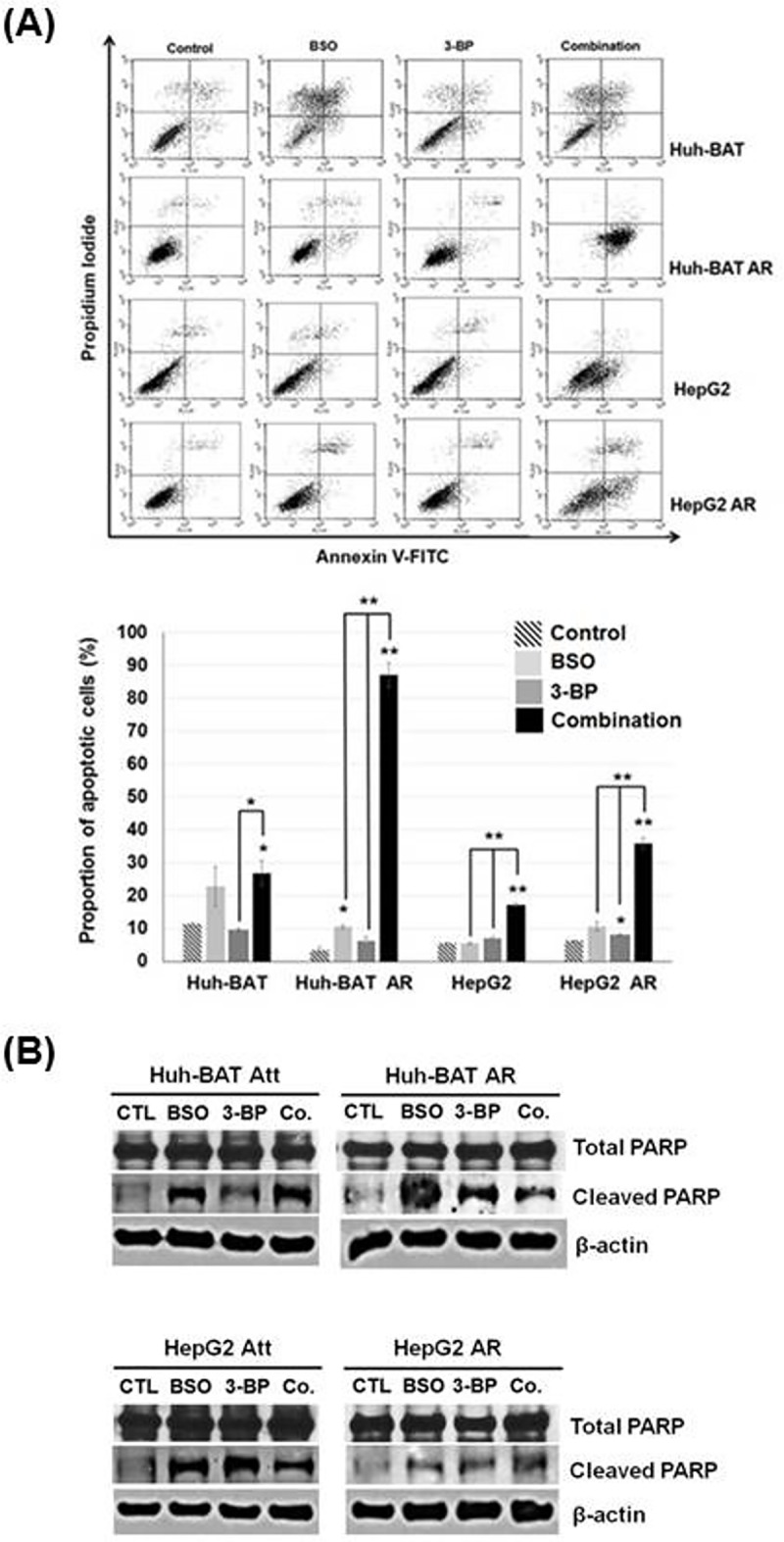
Apoptosis of Huh-BAT, HepG2, and the corresponding AR cells after 3-BP, BSO, and a combination treatment. (A) Apoptosis rates of Huh-BAT, HepG2, and the corresponding AR cells receiving the indicated treatments were evaluated by annexin V-FITC staining. The upper panel depicts the proportion of apoptotic cells, and the lower panel shows the quantitative results. (B) PARP expression in Huh-BAT, HepG2, and the corresponding AR cells receiving the indicated treatments was analyzed. At a combination treatment, 3-BP (40 μM) was treated for 48 hours at 24 hours after BSO treatment (200 μM) in the cells. *P<0.05; **P<0.01; ***P<0.001. Abbreviation: AR, anoikis-resistant; Att, attached; BSO, buthionine sulfoximine; Co., control; PARP, poly ADP-ribose polymerase; 3-BP, 3-bromopyruvate.

A combination treatment of 3-BP and BSO effectively induced apoptosis in both cancer cell types of attached and AR HCC cells compared to control (*P*<0.05 in Huh-BAT cells; *P*<0.01 in AR Huh-BAT cells; *P*<0.01 in HepG2 cells; *P*<0.01 in AR HepG2 cells) (Fig 6A). Cleaved PARP expression was increased in Huh-BAT, HepG2, and the corresponding AR cells when treated with 3-BP, BSO and a combination of both compared to the control (Fig 6B).

### Suppression of HCC invasion by a combination treatment of 3-BP and BSO

**Fig 7:**
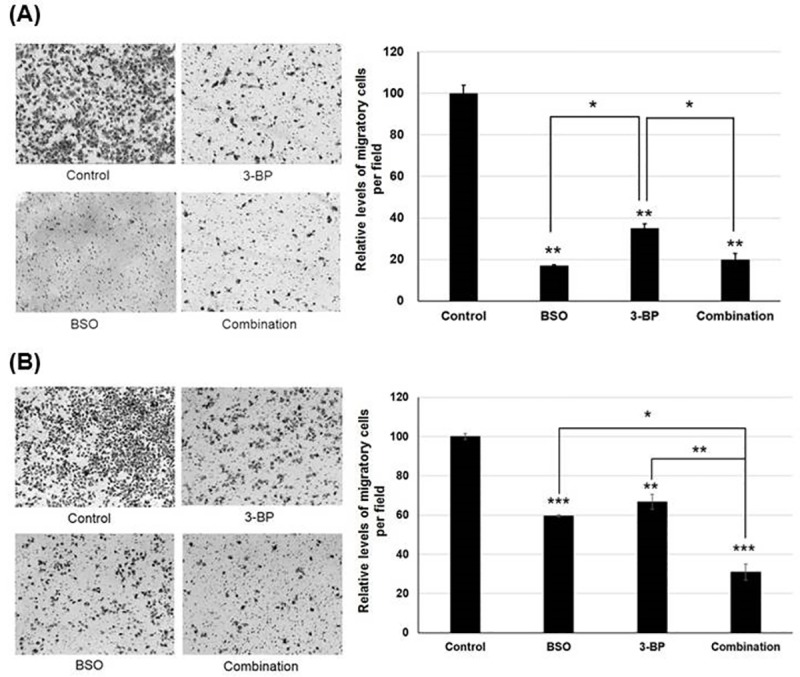
Suppression of HCC invasion by 3-BP, BSO, and a combination treatment. (A) Invasion capability of AR Huh-BAT cells significantly suppressed by BSO (200 μM), 3-BP (20 μM) and a combination by invasion assay using Boyden chambers (quantified at right panels). (B) Invasion capability of AR HepG2 cells significantly suppressed by BSO (200 μM), 3-BP (20 μM) and a combination by invasion assay using Boyden chambers (quantified at right panels). The concentrations of 3-BP (20 μM) and BSO (200 μM) which did not kill cancer cells were used in this assay. 3-BP and BSO was treated for 72 hours. In a combination treatment in this assay, 3-BP (20 μM) was treated for 72 hours with 24-hour pre-treatment of BSO (200 μM). *P<0.05; **P<0.01; ***P<0.001. Abbreviation: AR, anoikis-resistant; BSO, buthionine sulfoximine; 3-BP, 3-bromopyruvate.

3-BP, BSO, and the combined 3-BP with BSO treatment in AR Huh-BAT and AR HepG2 cells significantly suppressed cell invasion compared with the control (*P*<0.01, 3-BP; *P*<0.01, BSO; *P*<0.01, a combination treatment of 3-BP and BSO in AR Huh-BAT cells and *P*<0.01, 3-BP; *P*<0.001, BSO; *P*<0.001, a combination treatment of 3-BP and BSO in AR HepG2 cells). A combination treatment of 3-BP and BSO significantly suppressed cell invasion compared with 3-BP alone in AR Huh-BAT cells, and BSO or 3-BP alone in AR HepG2 cells (*P*<0.05, 3-BP, in AR Huh-BAT cells; *P*<0.01, 3-BP and *P*<0.05, BSO in AR HepG2 cells) (Fig 7A and 7B).

### Anti-tumor effect of a combination treatment of 3-BP and BSO in xenograft AR HCC models

Because BSO did not effectively suppress cell proliferation in in-vitro results in contrast to 3-BP or a combination treatment of 3-BP and BSO, we compared the tumor growth rates at treatment of 3-BP alone, 3-BP with BSO, and sorafenib which was used as a global standard treatment for patients with advanced HCC. Tumor buds were grown 10–12 days after implantation of AR Huh-BAT cells on the back of each mouse.

**Fig 8:**
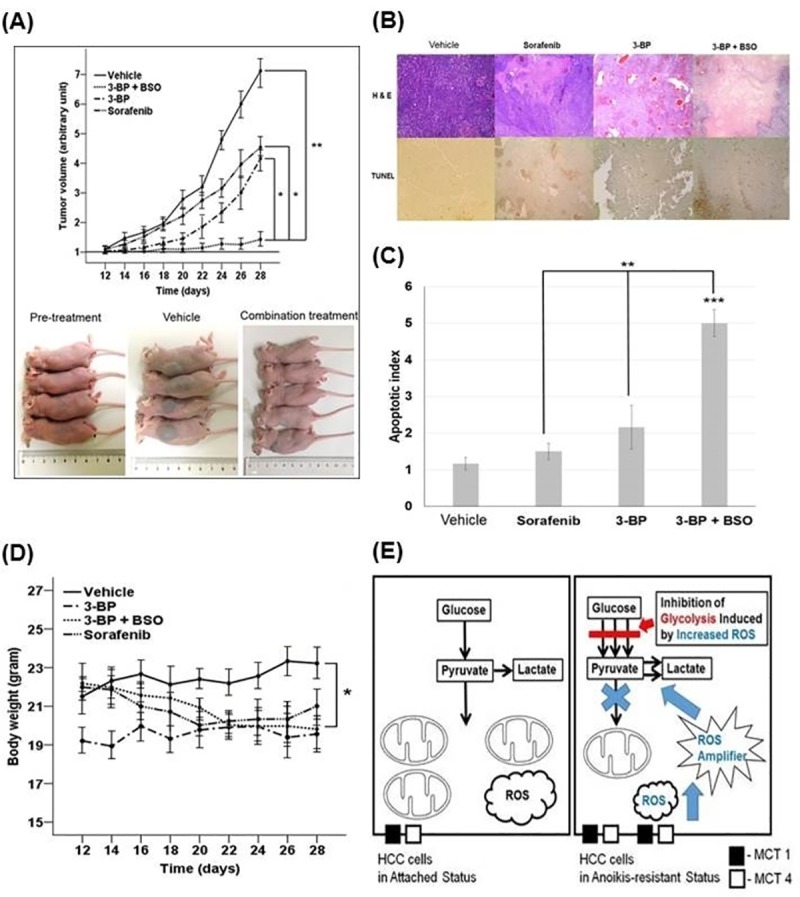
*In vivo* anti-tumor effects of 3-BP, sorafenib, and a combination treatment in xenograft nude mice bearing AR Huh-BAT cells. (A) Tumor growth rates in the combination treatment group were significantly lower than those in the control, sorafenib, or 3-BP treatment group (upper panel). Gross images of tumors before treatment, tumors from the control group, and tumors from the combination treatment group are shown (lower panel). (B) *In vivo* demonstration of the apoptosis-inducing efficacy in the control, 3-BP, sorafenib, and combination treatment group was shown: H & E and TUNEL staining of tumor tissues in the control, sorafenib, 3-BP, and combination-treated mice (×40 magnification). (C) TUNEL-positive cell percentages (apoptotic index) were determined in six different high power (×400 magnification) fields. (D) There was a significant difference in body weight between the control group and the combination treatment group (*P*<0.05). (E) After matrix detachment, anoikis-resistant cancer cells decrease intracellular ROS levels through inducing enzymes involved in the glycolysis and antioxidant systems for their survival. The Warburg effect can be modulated by increased intracellular ROS levels. Increased ROS levels induce HK II expression and make cancer cells sensitive to 3-BP treatment, and thereby promote cell death via ROS-mediated apoptosis (the black box indicates monocarboxylate transporter-1, and the white box indicates monocarboxylate transporter-4). *P<0.05; **P<0.01; ***P<0.001. Abbreviation: AR, anoikis-resistant; BSO, buthionine sulfoximine; 3-BP, 3-bromopyruvate.

A combination treatment of 3-BP and BSO effectively suppressed tumor growth as compared to other treatments. Tumor growth rates in the combination group were significantly lower than those in the control, sorafenib, and 3-BP treatment alone groups (*P*<0.01, *P*<0.05 and *P*<0.05, respectively) (Fig 8A). Although the 3-BP or sorafenib treatment alone groups tended to have lower tumor growth rates than the control group, there were no significant differences in tumor growth rates between the 3-BP and sorafenib treatment alone groups. There were no significant differences in the growth rates of the tumors between the control group and the 3-BP or sorafenib treatment alone groups (Fig 8A). The apoptotic index was significantly higher in the combination group compared to the other groups (*P*<0.001, control; *P*<0.01, the sorafenib treatment group; *P*<0.01, the 3-BP treatment group) (Fig 8B and 8C). The percentage of TUNEL-stained cells in the 3-BP or sorafenib treatment alone groups was not significantly different from that of the control group. There was a significant difference in body weight between the control group and the combination treatment group (*P*<0.05) (Fig 8D).

## Discussion

Five important findings emerged from this study: 1) expression of key enzymes involved in glycolysis, antioxidant systems, and EMT, such as HK II, p-PDH, MCT-1, rGCS, and Snail, was upregulated upon matrix detachment; 2) compared to attached HCC cells, AR HCC cells significantly increased lactic acid production and decreased ROS generation; 3) AR HCC cells showed chemoresistance to conventional chemotherapy agents, particularly sorafenib, and had higher tumor growth rates than attached HCC cells in animals; 4) high intracellular ROS levels accelerated glycolysis rates via HK II induction; 5) although BSO did not effectively suppress cancer cell proliferation, a combination treatment of 3-BP and BSO potently suppressed the AR HCC and attached cell proliferation rates via apoptosis and inhibited tumor growth compared to 3-BP or sorafenib treatment alone in a xenograft mouse model bearing AR HCC cells: BSO played a role for a booster to 3-BP effects.

Our results demonstrated that a combination treatment of 3-BP and BSO effectively suppressed growth of AR HCC cells via apoptosis. Increased ROS levels after BSO treatment stimulated the glycolytic pathway via HK II induction. It could be explained by that the cancer cells pre-treated with BSO potentiated the Warburg effect to evade excess ROS production from BSO treatment. Cells that were less dependent on mitochondrial oxidation with high HK II expression and increased generation of NADPH via the PPP could be more sensitive to 3-BP treatment compared to cells not pre-treated with BSO (Fig 8E). In AR HCC cells, expression of MCT-1, a gateway for 3-BP, was increased as compared to attached cells. This result reflects that intracellular glutathione levels can play a more important role to anti-tumor effects of 3-BP than higher expression of a 3-BP gateway such as MCT-1 because anti-tumor effects of 3-BP were decreased in spite of high MCT-1 expression in AR HCC cells.

This study has clinical implications as this strategy can target AR HCC cells, initiative cells for microscopic metastasis. Given there have been no effective therapies in patients with advanced HCC who showed sorafenib resistance, a combination treatment of 3-BP and BSO can be an effective alternative to suppress tumor growth. Furthermore, this strategy also suggests possibilities of adjuvant treatments for the patients who underwent curative treatments because early recurrence with a multinodular pattern and/or portal vein invasion after resection occurs due to microvascular invasion of HCC into normal liver parenchyma.

Previous studies reported that increased ROS levels can facilitate tumor growth, invasion, and angiogenesis [34]. A major implication of our findings is that antioxidants may not be beneficial during initiation of metastasis. ROS levels below the toxic threshold stimulating apoptosis activate signaling pathways, such as Src, PI3K, NF-kB, and HIF, that may increase cell proliferation under mild oxidative conditions [35]. In contrast, high ROS levels above the toxic threshold required for signaling may cause strong oxidative damage that can result in death in cancer cells [36]. Therefore, it would be important to deliver high doses of BSO to amplify intracellular ROS levels to induce apoptosis at the tumor site. In contrast to normal cells, cancer cells show a shift in redox dynamics with high ROS production and elimination to maintain the ROS levels below the toxic threshold [36, 37]. In previous reports, the potent ROS enhancer piperlongumine demonstrated potent anti-tumor effects on HCC cells via ROS accumulation [38].

There are three explanations regarding the propose mechanisms of synergistic effect of 3-BP and BSO. First, BSO can potentiate anti-tumor effect of 3-BP by suppressing the antagonist to 3-BP, i.e., GSH. Previous studies showed that 3-BP occurred a significant decrease in GSH concentrations in multiple myeloma cells [39]. This phenomenon was occurred by the 3-BP-GSH binding to inactivate 3-BP [40]. The enzyme that has the ability to catalyze the conjugation of the reduced form of GSH to the substrates is glutathione S-transferase (GST). Previous study showed that the GST gene expression increases after 3-BP exposure to multiple myeloma cells [39]. In addition, it was reported that 3-BP alters the level of the expression of genes encoding other crucial enzymes involved in GSH metabolism such as rGCS and glutathione synthetase [39]. Given that GSH plays a role for attenuating oxidative stress in the cancer cell, any compounds which potentiate oxidative stress can reduce intracellular GSH levels. Thereby, the reduced levels of GSH by other agents such as ROS enhancers or BSO can indirectly potentiate the anticancer effect of 3-BP. Second, in this study, intracellular ROS levels were significantly increased when cancer cells were treated with BSO and 3-BP compared to those treated with 3-BP alone. Potent anticancer effect of BSO and 3-BP can be explained by enhanced ROS levels induced by both compounds. Third, ROS modulation by BSO potentiated HK II expression in the cancer cells, which can make the cells to be sensitive to 3-BP. The findings in this study which increased ROS levels potentiated glycolysis were in line with the previous studies: increased intracellular ROS levels reduced oxidative phosphorylation and favored aerobic glycolysis such as HK II activation in the cancer cells [41–44].

Accelerated glycolysis rates enhance cell proliferation and anti-oxidant capacity by activating the PPP and keeping pyruvate away from mitochondrial oxidation to avoid the generation of excess ROS. It indicates that metabolic reprogramming can increase anti-oxidant capacity that favors survival of cancer cells against high ROS levels [45]. Moreover, increased ROS levels can reduce oxidative phosphorylation via various mechanisms including stabilization of HIF1-α and inactivation of protein tyrosine phosphatases, thereby favoring aerobic glycolysis and proliferation [41–44]. Oxidative stress induced by any agents or chemotherapy can aggravate metabolic reprogramming in cancer cells to favor glycolysis, and it can make cancer cells to be more sensitive to potent glycolytic inhibitor such as 3-BP.

The results in this study suggest that changes in ROS levels can affect metabolic reprogramming, and more importantly that dual inhibition of anti-oxidant system and glycolysis can effectively suppress tumor growth in anoikis-resistant cancer cells. This strategy based on targeting mutual interaction between ROS and glycolysis can be applied to cancers, which had characteristics of early metastasis, aggressive growth, or chemoresistance such as HCC, pancreatic cancer, and breast cancer unresponsive to hormone therapy or chemotherapy.

This study had some limitations. First, BSO treatment could not specifically target cancer cells. As ROS homeostasis is important for normal cell senescence, renewal, and inflammation, adverse effects of increased oxidative stress by BSO treatment should be considered. In this study, body weights of mice treated with a combination of 3-BP and BSO were significantly decreased compared to those of the single treatment or the control groups, indicating the toxicity of oxidative stress potentiation by a combination treatment of 3-BP and BSO. Therefore, to minimize toxicity of BSO, accurate delivery of ROS enhancers to the tumor sites should be investigated. Second, in this study, there were no comparable groups in which animal were treated with BSO alone when anti-tumor effects in animals treated with a combination treatments of 3-BP and BSO were compared with those in animals treated with 3-BP alone or sorafenib. However, given the anti-tumor effect of BSO in vitro was negligible, not effective as compared to that of 3-BP or sorafenib, animal group treated with BSO alone might be not necessary to evaluate the anti-tumor effect of BSO in vivo.

In conclusion, human AR HCC cells showed chemoresistance, an aggressive growth phenotype, and higher glycolysis, metastatic potential, and antioxidant systems compared to attached HCC cells. A combination treatment of 3-BP and BSO effectively suppressed AR HCC and attached cell growth. The results suggest an alternative therapy for HCC patients with intra- or extra-hepatic metastasis who show a poor response to sorafenib treatment.

## Supporting information

S1 Appendix (DOCX)

## Abbreviations

AR: anoikis-resistant
BSO: buthionine sulfoximine
CTL: control
EMT: epithelial–mesenchymal transition
GSH: glutathione
HCC: hepatocellular carcinoma
HK II: hexokinase II
MCT-1: monocarboxylate transporter-1
MnSOD: manganese superoxide dismutase
PARP: Poly (ADP-ribose) polymerase
p-PDH: phosphorylated pyruvate dehydrogenase
rGCS: gamma-glutamylcysteine synthetase
ROS: reactive oxygen species
TUNEL: terminal deoxynucleotidyl transferase-mediated dUTP nick end labeling
3-BP: 3-bromopyruvate
