# Supplementary material for: 3-bromopyruvate and buthionine sulfoximine effectively kill anoikis-resistant hepatocellular carcinoma cells
Source: PLoS One. 2017 Mar 31;12(3):e0174271. doi: 10.1371/journal.pone.0174271 (PMC5376082; doi:10.1371/journal.pone.0174271)
Supplement: S1 Appendix — (DOCX) [file pone.0174271.s001.docx]

**S1A Appendix. Cell culture and reagents**

For experiments comparing the attached and AR HCC cells, 5×10^5^ cells were seeded in appropriate culture dishes, grown for 48 hours, harvested, treated with diluted trypsin-EDTA, and analyzed as indicated. In a set of experiments, cells were incubated for 72 hours with media containing each compound: 40 µM 3-BP (Sigma, St. Louis, MO), 200 µM BSO (Santa Cruz, Dallas, Texas), 3 µM sorafenib (LC Laboratories, Woburn, MA), 1 µM 5-fluorouracil (5-FU) (Sigma, St. Louis, MO), 10 µM cisplatin (Sigma, St. Louis, MO), and 10 nM doxorubicin (Sigma, St. Louis, MO).

The Cell Titer 96 Aqueous One Solution cell proliferation assay (Promega, Madison, WI, USA) was used to measure cell viabilities. In this assay, dehydrogenase enzymes convert the colorimetric MTS reagent [3,4-(5-dimethylthiazole-2-yl)-5-(3-carboxymethophenyl)-2-(4-sulfophenyl)-2H-tetrazolium salt] into soluble formazan in metabolically active proliferating cells. After each treatment, 20 μL of dye solution was added to each well of a 96-well plate and incubated for 2 hours. Absorbance at 490 nm was measured with an ELISA plate reader (Molecular Devices, Sunnyvale, CA, USA)

**S1B Appendix. Western blot analysis**

Anti-HK II (Santa Cruz, Dallas, Texas), rGCS (Santa Cruz, Dallas, Texas), phosphorylated pyruvate dehydrogenase (p-PDH) (Abcam, Cambridge, MA), Snail (Cell Signaling Technology, Danvers, MA), E-cadherin (Cell Signaling Technology, Danvers, MA), Poly (ADP-ribose) polymerase (PARP), cleaved PARP (Cell Signaling Technology, Danvers, MA), MCT-1 (Santa Cruz, Dallas, Texas), and β-actin (Santa Cruz, Dallas, Texas) were purchased.

**S1C Appendix. Invasion assay**

We used a low dose of 20 μM, 3-BP to prevent rapid apoptosis by a combination treatment of 40 μM, 3-BP and 200 μM, BSO, and to measure cell invasion ability for longer duration. AR Huh-BAT and AR HepG2 cells were treated as indicated (vehicle control, 200 µM of BSO, 20 µM of 3-BP, or a combination treatment of 3-BP and BSO). DMEM containing 10% FBS was added to the chambers. Cells were incubated in a 24-well plate at 37°C with 5% CO_2_. After 24 hours, cells that had not migrated to the lower wells were removed from the upper chamber using clean cotton swabs, and cells that had moved to the lower surface of the filter were stained using a staining solution. Cell invasion was quantified by visual counting after being photographed. Experiments were performed in triplicate. Mean values for three random fields were obtained for each well.

**S1D Appendix. Measurement of intracellular lactic acid levels**

Intracellular lactic acid levels were measured using lactate assay kit (BioVision, Milpitas, CA). The assay was performed following the manufacturer’s instructions. For experiments with 40 µM of 3-BP or 200 µM of BSO, the cells were pretreated as described above. The relative concentrations were normalized to the cell number of each sample. Briefly, cells were homogenized in lactate assay buffer and deproteinized with 10 kDa spin filters to remove insoluble material. The lactate concentration was measured at 535/590 nm using a multiwall plate reader. Experiments were performed in triplicate.

**S1E Appendix. Measurement of intracellular glutathione levels**

Intracellular glutathione levels were quantiﬁed with a glutathione fluorometric assay kit (BioVision, Milpitas, CA) by following manufacture’s instruction. Brieﬂy, after cells were treated with 40 µM of 3-BP or 200 µM of BSO for 48 hours, 1ｘ10^6^ cells were homogenized in PCA on ice for 5 minutes and centrifuged at 13,000 g for 2 minutes. The supernatants were collected, and 20 µl of cold KOH was added to neutralize the samples. Neutralized samples were transferred to a 96-well plate for detection on a fluorescence plate reader equipped with fluorometric absorbance at 340/420 nm. Experiments were performed in triplicate.

**S1F Appendix. Detection of intracellular ROS production**

Intracellular ROS levels were determined using the ROS/RNS assay kit (Abcam, Cambridge, MA) which was based on DCFH probe. Cells were treated with 40 µM of 3-BP or 200 µM of BSO and collected in PBS. Cell lysates were added into a 96-well black plate. Then, catalyst was added into all test wells. Samples were mixed well and incubated 5 minutes at room temperature. The DCFH solution was added into all test wells and incubated at room temperature for 30 minutes. The fluorescence was read at 480 nm excitation/530 nm emission. To visualize, cells were dyed through fluorescent dye reagents. Upon staining, the fluorescent products generated by the dye were visualized using a wide-field fluorescence microscope equipped with standard red fluorescent cubes (Ex/Em=650/670 nm). Experiments were performed in triplicate.

**S1G Appendix. Apoptosis determination**

HCC cells were cultured and divided into 4 groups according to the treatment: (i) control, (ii) 3-BP alone, (iii) BSO alone, and (iv) a combination treatment of 3-BP and BSO. After trypsinization, the cells were incubated with 8 µl annexin V-fluorescein isothiocyanate (BD Biosciences, Franklin Lakes, NJ) and 5 µl propidium iodide (Sigma, St. Louis, MO) for 15 minutes in darkness. Degree of apoptosis was analyzed by fluorescence activated cell sorting. The proportion of stained cells in each quadrant was quantified with CellQuest software (BD Biosciences, Franklin Lakes, NJ). Expression of total and cleaved PARP was compared according to the treatment: (i) control, (ii) 3-BP alone, (iii) BSO alone, and (iv) a combination treatment of 3-BP and BSO. Experiments were performed in triplicate.

**S1H Appendix. Statistical analysis**

All experimental results represent at least 3 independent experiments using cells from a minimum of three separate isolations. Data were expressed as means with SEM. *P* values were calculated with Student’s paired *t* tests, or repeated measures ANOVA (SPSS, version 19.0, SPSS, Inc., Chicago, IL, USA). *P*<0.05 was considered statistically significant.
